# Extraction, Physicochemical Properties, and *In Vitro* Antioxidant Activities of Chondroitin Sulfate from Bovine Nose Cartilage

**DOI:** 10.1155/2024/6328378

**Published:** 2024-05-17

**Authors:** Chan Shi, Yuxuan Deng, Xin An, Yuan Chen, Xingang Lv, Qian Liu

**Affiliations:** College of Food Science and Technology, Northwest University, Xi'an 710069, China

## Abstract

Beef is an important high-nutrition livestock product, and several byproducts, such as bovine cartilage, are produced during slaughter. To effectively utilize these agricultural and pastoral byproducts, combined (trypsin-papain) enzymolysis and cetylpyridine chloride purification methods were used to obtain chondroitin sulfate (CS) from the nasal cartilage of Shaanxi Yellow cattle. The effects of pH, temperature, and time on the CS yield during enzymatic hydrolysis were investigated, and the CS extraction process was optimized using response surface methodology. The best yield of CS was 21.62% under the optimum conditions of pH 6.51, temperature of 64.53°C, and enzymolysis time of 19.86 h. The molecular weight of CS from Shaanxi cattle nasal cartilage was 89.21 kDa, glucuronic acid content was 31.76 ± 0.72%, protein content was 1.12 ± 0.03%, and sulfate group content was 23.34 ± 0.08%. The nasal cartilage CS of the Yellow cattle showed strong DPPH•, •OH, and ABTS^+^• radical scavenging abilities and ferrous reduction ability in the experimental concentration range. This study could contribute to “turn waste into treasure” and improve the comprehensive utilization of regional characteristic biological resources.

## 1. Introduction

Beef is an important high-nutritional livestock product [1]. China has abundant grasslands and beef production resources, including Yellow cattle that have strong adaptability, coarse feeding resistance, and good grazing performance. They can survive extreme heat during summer, as well as feed, graze, and ruminate normally. Several byproducts, such as bovine cartilage, are produced during the slaughter of beef cattle [2]. It is socioeconomically and environmentally beneficial to convert waste into valuables and realize the high-value utilization of the Yellow cattle cartilage, which is considered a waste in agriculture and animal husbandry.

Chondroitin sulfate (CS), which consists of N-acetyl-D-galactosamine sulfate and D-glucuronic acid, is a type of glycosaminoglycan that is generally found in the connective tissues of mammals such as sheep, cattle, and pigs, as well as in sea cucumbers, sharks, and squid [3–5]. Numerous studies have shown that CS has strong antioxidant, immune regulatory, anticancer, and other biological activities and plays important roles in cell transfer, differentiation, proliferation, recognition, and tissue formation [6, 7]. CS proteoglycans have a specific affinity for low-density lipoprotein (LDL) and can reduce local cholesterol deposition by regulating the recruitment of immunoglobulins, amyloids, and cholesterol around macrophages [8, 9]. CS is considered the best drug for preventing and treating cardiovascular diseases and arthropathy [10]. Several formulations and specifications for CS with few side effects that are safe for long-term use are currently in the market. In addition, CS absorbs and retains moisture, strengthens connective tissue, and improves skin cell metabolism [11].

In this study, CS was extracted from the bovine nasal cartilages of Shaanxi Yellow cattle by a response surface methodology- (RSM-) optimized combined enzymolysis method and characterized by high-performance liquid chromatography (HPLC), high-performance gel permeation chromatography (HPGPC), m-hydroxybiphenyl method, TCA kit method, Fourier transform infrared (FTIR) spectroscopy, and nuclear magnetic resonance (NMR) spectroscopy. The antioxidant activities of CS were further studied, including the ferrous reduction ability and scavenging ability of 1,1-diphenyl-2-pyridinehydrazide radical (DPPH•), hydroxyl radical (•OH), and 2,2′-azinobis-3-ethylbenzthiazoline-6-sulphonate radical (ABTS^+^•). This study is aimed at “turning waste into treasure” and improving the comprehensive utilization of biological resources, thus improving the income and living standards of the farmers and realizing the coexistence of social, economic, and ecological benefits.

## 2. Materials and Methods

### 2.1. Experimental Materials and Reagents

The bovine nose cartilage of Shaanxi cattle was purchased from the Xi'an Muslim Quarter, and the nose cartilage of Jiangsu cattle was purchased from the Lianyungang market. CS standards (95%, CAS # 9007-28-7) and 1-phenyl-3-methyl-5-pyrazolone were purchased from Macklin Biochemical Co., Ltd. (Shanghai, China). Papain (800 U/mg, CAS # 9001-73-4) was purchased from Shanghai Yuanye Biotechnology Co., Ltd. (Shanghai, China). Trypsin (≥280 USP • E/mg, CAS # 9002-07-7) was purchased from BioFroxx (Einhausen, Germany). Monosaccharide (mannose, rhamnose, glucuronic acid, galacturonic acid, glucose, N-acetylgalactose, galactose, xylose, arabinose, and fucose), and dextran standards (1, 5, 12, 25, 50, 80, 150, and 270 kDa) were purchased from Sigma-Aldrich Trading Co., Ltd. (St. Louis, MO, USA). Potassium sulfamate was purchased from Shanghai Acmec Biochemical Co., Ltd. (Shanghai, China). Glucuronic acid, H_2_O_2_, and ascorbic acid (Vc) were purchased from Chengdu Kelong Chemical Co., Ltd. (Chengdu, China). m-Hydroxybiphenyl was purchased from Shanghai Sanaisi Reagent Co., Ltd. (Shanghai, China). Chlorohexadecylpyridine was provided by the Tianjin Guangfu Institute of Fine Chemicals (Tianjin, China). Sodium hydrogen tetraborate and sodium tetraborate were provided by the Tianjin Fuchen Chemical Reagent Factory (Tianjin, China). L-Cysteine, phenol, FeSO_4_, FeCl_3_·6H_2_O, trichloroacetic acid, trifluoroacetic acid, dichloromethane, and glacial acetic acid were provided by the Tianjin Damao Chemical Reagent Factory (Tianjin, China). 2,3,5-Chlorotriphenyltetrazolium (TTC) was purchased from Shanghai Lanji Technology Development Co., Ltd. (Shanghai, China). DPPH• and ABTS^+^• reagents were purchased from DIYIBio (Beijing, China). K_2_S_2_O_8_ was purchased from Xilong Scientific Co., Ltd. (Guangdong, China). Salicylic acid was purchased from Tianjin Kemio Chemical Reagent Co., Ltd. (Tianjin, China). Barium chloride was purchased from Tianjin Tianli Chemical Reagent Co., Ltd. (Tianjin, China). All other reagents were purchased from China and were of HPLC-grade or the highest commercially available grade.

### 2.2. Extraction and Purification of CS

The bovine nasal cartilage was washed to remove the adhering meat and impurities and then defatted with acetone, air-dried, and pulverized for subsequent use [12, 13]. Bovine nasal cartilage powder (10 g) was weighed; then, 0.01 M cysteine buffer and 0.01 M EDTA were added at a ratio of 1 : 10. After adjusting the pH to 6.5, 1.5% compound enzyme solution (papain : trypsin =1 : 2, *w*/*w*) was added and reacted in a 65°C water bath for 24 h. The solution was centrifuged at 3,000 rpm for 10 min, and the supernatant was added with 1.25× volume of ethanol to precipitate at 4°C for 12 h. The precipitate was resuspended in distilled water and reprecipitated with 2× volume of ethanol (containing 0.5% sodium acetate). The precipitate was centrifuged for 10 min at 3,000 rpm then resuspended in 200 mL of 0.5 M NaCl solution (containing 5% cetylpyridine chloride (CPC)). The mixture was incubated overnight at 25°C. After centrifugation at 3,000 rpm for 10 min, the precipitates were resuspended in 100 mL of 2 M NaCl : ethanol (100 : 15, *v*/*v*), and the centrifugation process was repeated three times using ethanol. The precipitate was resuspended in distilled water and then dialyzed under running water for three days. The solution was concentrated under reduced pressure and freeze-dried to obtain CS samples.

#### 2.2.1. Single-Factor Experiments

To improve the CS yield, the enzymatic hydrolysis conditions (pH, temperature, and time) were optimized by single-factor experiments.

*(1) Effect of Enzymolysis pH on the CS Yield*. Bovine nasal cartilage powder (10 g) was weighed and extracted. The enzymolysis pH were set at 5.0, 6.5, and 8.0, enzymolysis temperature was set at 65°C, and enzymolysis time was set at 24 h. After the CPC was purified, dialyzed, and freeze-dried, the sample weight was measured to determine the effect of the enzymolysis pH on the CS yield.

*(2) Effect of Enzymolysis Temperature on the CS Yield*. Bovine nasal cartilage powder (10 g) was weighed and extracted. Enzymolysis temperatures were set at 37°C, 51°C, and 65°C, enzymolysis pH was set at 6.5, and enzymolysis time was set at 24 h. After the CPC was purified, dialyzed, and freeze-dried, the sample weight was measured to investigate the effect of enzymolysis temperature on the CS yield.

*(3) Effect of Enzymolysis Time on the CS Yield*. Bovine nasal cartilage powder (10 g) was weighed and extracted. Enzymolysis times were set at 12, 24, and 36 h, enzymolysis pH was set at 6.5, and enzymolysis temperature was set at 65°C. After the CPC was purified, dialyzed, and freeze-dried, the sample weight was measured to investigate the effect of enzymolysis time on the CS yield.

#### 2.2.2. Response Surface Optimization

The ranges of independent variables, enzymatic hydrolysis pH (*X*_1_), enzymatic hydrolysis temperature (*X*_2_), and enzymatic hydrolysis time (*X*_3_) were determined by single-factor experiments. Three levels and three factors Box–Behnken design (BBD) were used to improve the parameters of CS extraction using the compound enzyme method. All variables were fixed at levels −1, 0, and 1. The natural and coded values of the independent variables used in the BBD are listed in Table 1. The experimental data were analyzed by multiple regression analysis. In the regression analysis, the independent and response variables were fitted to a quadratic polynomial model in the following general form: *Y* is the response variable (CS yield), *X*_*i*_ and *X*_*j*_ are the independent variables, and *X*_*i*_^2^ and *X*_*i*_*X*_*j*_ are the quadratic and interaction terms of the variables, respectively. Design-Expert 12.0 was used to generate the experimental design, regression model, statistical analysis, and optimization of the enzymolysis extraction conditions.

### 2.3. Determination of Physicochemical Properties of CS

The monosaccharide composition of CS was determined using the PMP precolumn derivatization method [14]. Briefly, 2 mg of CS was dissolved in 1.7 mL of distilled water and then added with 0.3 mL of trifluoroacetic acid. The mixture was incubated at 121°C for 2 h. During rotary evaporation, 2 mL of double-distilled water was added and repeatedly pumped under reduced pressure to remove the trifluoroacetic acid, and the neutrality of the solution was verified using a pH test paper. NaOH (200 *μ*L 0.3 mol/L) and PMP methanol reagent (200 *μ*L 0.5 mol/L) were added, and then, the mixture was thoroughly mixed by shaking and incubated in a 70°C water bath for 1.5 h. The solution was then neutralized with 0.3 mol/L HCl, cooled to room temperature, and added with equal volume of dichloromethane for extraction. The mixture was thoroughly mixed and centrifuged at 10,000 rpm for 3 min. The C18 solid-phase extraction column was activated by washing with 3 mL of 100% acetonitrile and 15 mL of double-distilled water for equilibration. The pH was adjusted to 4.0 by adding glacial acetic acid to the supernatant. An equal volume of dichloromethane was added thrice for extraction and then concentrated under reduced pressure and rotary evaporation at 39°C to dryness. Double-distilled water (1 mL) was added to redissolve the sediment, which was then passed through a C18 extraction column. Double-distilled water (20 mL) was added for desalination, and 3 mL of 25% acetonitrile was added for elution. The eluate was filtered using a 0.22 *μ*m syringe filter membrane and then placed into a liquid-phase vial for storage at −20°C for subsequent use. The monosaccharide standard was PMP precolumn derivatized according to the above procedure, and the samples were injected sequentially. The HPLC conditions were as follows: RP-HPLC: 4.6 × 250 mm SinoChrom ODS-BP C18 (5 *μ*m, Dalian, Park Jung Su) chromatographic column; mobile phase A: acetonitrile and phase B: 0.02 mM ammonium acetate buffer; elution: 0–25 min, 83% B, 25–50 min, 85% B, and 50–130 min, 87% B; flow rate: 0.8 mL/min; injection volume: 15 *μ*L/sec; elution time: 130 min; column temperature: 30°C; and detection wavelength: 245 nm.

The average molecular weight of the polysaccharides was determined by HPGPC [15]. The TSK-Gel G4000SWXL dextran gel chromatography column was used, and the detector was a differential refractive index detector. The conditions were as follows: mobile phase: phosphate buffer salt solution with pH 6.0; flow rate: 0.3 mL/min; and sample volume: 20 *μ*L. The average molecular weight of the samples was calculated from the retention time of dextran standards with different molecular weights.

The infrared spectrum of CS in the range of 4,000–400 cm^−1^ was recorded using the EQUINOX 55 FTIR spectrophotometer (Bruker, Germany). CS and standard samples were prepared using pressed KBr particles.

The samples were dissolved in 0.5 mL of D_2_O to prepare a final concentration of 40 mg/mL. 1D-NMR (^1^H-NMR, ^13^C-NMR) was performed by Sanshu Biotech. Co., Ltd. (Shanghai, China) using the Bruker AVANCE NEO 500 M spectrometer system (Bruker, Rheinstetten, Germany) operating at 25°C and 500 MHz.

### 2.4. In Vitro Antioxidant Capacity Analysis

#### 2.4.1. DPPH• Scavenging Assay

This method was based on a previous study investigating the DPPH• scavenging ability of CS [16]. The same amount of DPPH methanol solution (0.02 mM) was added to the CS samples at different concentrations (0.75–30 mg/mL) and allowed to react at room temperature in the dark for 30 min. Using Vc as a positive control, the absorbance at 517 nm was measured using an enzyme-labeled instrument. The DPPH• scavenging rate of the CS samples was calculated as follows:

(1) $$\begin{array}{c} C=\left(1-\frac{A_{i}-A_{j}}{A-A_{0}}\right)\times 100\%, \end{array}$$

where *A*_*i*_ is the absorbance of the experimental group, *A*_*j*_ is the absorbance of the control group (equal volume of methanol solution mixed with CS sample), *A* is the absorbance of the blank group (equal volume of methanol mixed with DPPH methanol solution), and *A*_0_ is the absorbance of methanol.

#### 2.4.2. •OH Scavenging Assay

The Fenton method for •OH determination was followed, with slight modifications [17]. FeSO_4_ (200 *μ*L, 1.8 mmol/L), salicylic acid/ethanol solution (150 *μ*L, 1.8 mmol/L), and H_2_O_2_ (10 *μ*L, 0.03%) were added to 100 *μ*L of CS solution with different concentrations (0.75–30 mg/mL). After thoroughly mixing by vortex oscillation, the solution was incubated in a 37°C water bath for 30 min and centrifuged at 3,000 rpm for 20 min. The absorbance of the supernatant was measured at 510 nm using an ultraviolet-visible spectrophotometer, and Vc was used as the positive control. The •OH clearance of the samples was calculated according to the following formula:

(2) $$\begin{array}{c} C=1-\frac{A_{1}-A_{2}}{A_{0}}\times 100\%, \end{array}$$

where *A*_1_ is the absorbance of the experimental group, *A*_2_ is the absorbance of the control group (using ultrapure water instead of FeSO_4_, salicylic acid-ethanol solution, and H_2_O_2_), and *A*_0_ is the absorbance of the blank group (using ultrapure water instead of the sample solution).

#### 2.4.3. ABTS^+^• Scavenging Assay

The ABTS^+^• scavenging assay was performed as previously described, with minor modifications [18]. To prepare the ABTS^+^• stock solution, 7 mmol/L ABTS^+^• and 7.35 mmol/L K_2_S_2_O_8_ were mixed at a 2 : 1 ratio and reacted in the dark at 25°C for 16 h. CS sample solutions (60 *μ*L) of different concentrations (0.75–30 mg/mL) were added to 300 *μ*L of ABTS^+^• working solution (ABTS^+^• free radical storage solution diluted with anhydrous ethanol, OD 734 nm was within 0.70 ± 0.02). The solution was vortexed for uniform mixing and reacted in the dark at 25°C for 6 min. Absorbance was measured at 734 nm. Vc was used as the positive control, and the removal rate of ABTS^+^• by the sample was calculated according to the following formula.

(3) $$\begin{array}{c} C=\left(1-\frac{A_{1}-A_{2}}{A_{0}}\right)\times 100\%, \end{array}$$

where *C* is the ABTS^+^• scavenging rate, *A*_1_ is the absorbance of the test group, *A*_2_ is the absorbance of the control group (using ultrapure water instead of ABTS^+^• working solution), and *A*_0_ is the absorbance of the blank group (using ultrapure water instead of sample solution).

#### 2.4.4. Reducing Power Assay

The ferrous reduction capability was determined, with minor modifications [19]. To prepare the ferric reducing antioxidant power (FRAP) working solution, 300 mmol/L sodium acetate buffer pH 3.6, 10 mmol/L TPTZ, and 20 mmol/L FeCl_3_·6H_2_O were mixed at 10 : 1 : 1.

FeSO_4_ standard solution (60 *μ*L, 0–500 mol/L) and TPTZ working solution (300 *μ*L) were added, mixed by vortex shaking, and incubated in the dark for 30 min. The absorbance was measured at 593 nm. The reducing ability of ferrous ions was calculated according to formula (4), and a standard curve was constructed with the FeSO_4_ concentration and clearance rate as the horizontal and vertical coordinates, respectively. The FRAP value of the CS sample was expressed as the amount of FeSO_4_ required to achieve the same absorbance.

(4) $$\begin{array}{c} C=A_{1}-A_{2}-A_{0}, \end{array}$$

where *A*_1_ is the absorbance value of the test group, *A*_2_ is the absorbance value of the control group (using ultrapure water instead of the TPTZ working solution), and *A*_0_ is the absorbance value of the blank group (using ultrapure water instead of the FeSO_4_ standard solution).

### 2.5. Statistical Analysis

Each experiment was repeated at least thrice, and the data were expressed as the mean ± standard deviation (*X* ± SD). Significant differences between the mean values were determined by one-way analysis of variance (ANOVA) using SPSS 19.0. The mean values were considered statistically significant at *P* < 0.05.

## 3. Results and Discussion

### 3.1. Analysis of Single-Factor Experiment

**Figure 1:**
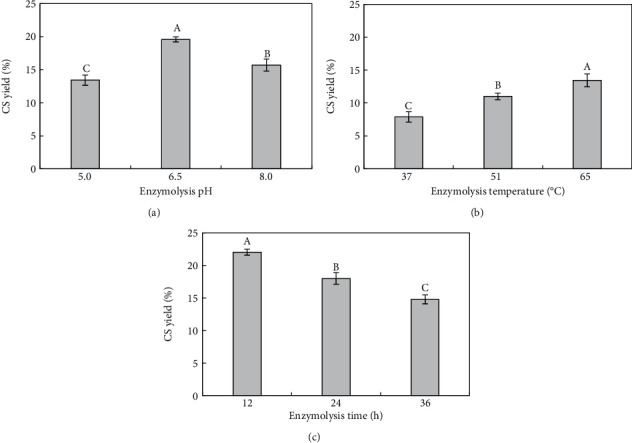
Effects of extraction factors on the yields of bovine nose cartilage chondroitin sulfate: (a) enzymatic hydrolysis pH; (b) enzymatic hydrolysis temperature; (c) enzymatic hydrolysis time. Results were expressed as *mean* ± *SD* (*n* ≥ 3). Different superscript letters indicate the significant difference (*P* < 0.05).

In this study, combined (trypsin-papain) enzymatic hydrolysis and CPC purification were used to extract CS from the bovine nasal cartilage of Shaanxi cattle, and the effects of pH, temperature, and time on the CS yield during enzymatic hydrolysis were investigated. The results of the single-factor experiments are shown in Figure 1.

As illustrated in Figure 1(a), the highest yield was obtained at pH 6.5. The dissociation state of the group was determined by the solution pH. When the enzyme activity was high, the enzymolysis speed and yield increased. Conversely, when the enzyme activity was inhibited, the extraction rate decreased. The pH also affected the degree of cell rupture and reduced the dissolution of active ingredients. The disturbance in the charge distribution directly affects enzymatic hydrolysis.

Figure 1(b) shows that the highest yield was obtained at 65°C enzymolysis temperature, and the yield gradually increased as the temperature increased. An increase in temperature accelerated the enzymatic reaction. At low temperatures, molecular diffusion is slow, and the enzyme did not function optimally, resulting in inadequate enzymolysis. On the other hand, enzymes may have a passivation effect at high temperatures, and the *β*-elimination reaction of the easily oxidized and degraded polysaccharide could be accelerated, resulting in the deepened color and decreased purity of the final products [20].

As illustrated in Figure 1(c), the highest yield was obtained after 12 h of enzymolysis. If the enzymolysis time was too short, the enzyme did not penetrate, the reaction with the substrate was incomplete, and the protein was deposited and remained intact. However, if the enzymolysis time is too long, some impurities change the enzyme structure and even inactivate the enzyme, resulting in a low polysaccharide yield.

### 3.2. Extraction Process Optimization of CS by RSM

RSM can analyze the interaction between the factors that affect the test response value, and statistical analysis shows the significance level of each factor. The advantage of RSM is the selection of representative experimental points, which is necessary to evaluate multiple variables and their interaction effects, thereby reducing the number of experimental groups [21]. The two most commonly used experimental designs for response surface optimization are the central composite design, which is suitable for multifactor and multilevel experimental designs, and BBD, which is more suitable for three-level experimental designs with few factors [22]. In this study, BBD was used to optimize the CS extraction process.

The natural and coded values of the BBD are presented in Table 1, and the design matrix and response values for the BBD experiments are presented in Table 2. The results showed that the CS yield ranged from 5.49 to 22.04%. The differences between the actual and predicted values are listed in Table 1. ANOVA and multiple regression analysis were conducted to analyze the experimental data. Considering the three factors (*X*_1_, *X*_2_, and *X*_3_), a quadratic model for the extraction rate of CS (*Y*) was established as follows:

(5) $$\begin{array}{c} Y\left(\%\right)=-0.5X_{1}+4.34X_{2}+0.35X_{3}+0.615X_{1}X_{2}+0.03X_{1}X_{3}-2.98X_{2}X_{3}-5.62X_{1}^{2}-2.78X_{2}^{2}-3.68X_{3}^{2}+19.59. \end{array}$$

The BBD of the experimental results optimized by ANOVA are presented in Table 3. The *P* value of the model was low (0.0023) and the *F* value was high (11.03), indicating that the regression model was significant. The effects of *X*_2_^2^ and *X*_2_*X*_3_ treatments were significant, whereas those of *X*_2_, *X*_1_^2^, and *X*_3_^2^ were extremely significant. The data and model were effective, and the model effectively predicted the response value, indicating that the experimental value of the BBD was reliable. *R* > 0.9 further indicates that the model has good credibility. The order of influence of the three factors on the yields was *X*_2_ > *X*_1_ > *X*_3_; the enzymolysis temperature was the most influential factor, followed by enzymolysis pH and time.

**Figure 2:**
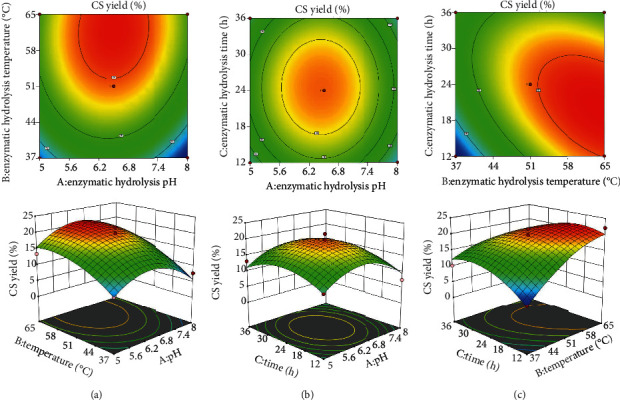
Response surface 3D plots and corresponding 2D contour plots of significant interaction terms: (a) *Y* = *f* (pH A, temperature B) response surface and contour map (time 24 h); (b) *Y* = *f* (pH A, time C) response surface and contour map (temperature 65°C); (c) *Y* = *f* (temperature B, time C) response surface and contour map (pH 6.5).

When examining the influence of the interactions among the three independent variables, the values of the other two variables were set as codes, and there were three interactions among the independent variables. The generated response surface and contour maps are shown in Figure 2. When the value was small, the response surface curve was steep, indicating that the impact on the CS yield was relatively significant. When the value was large, the response surface curve was flatter, and the effect on the yield was smaller.

Figure 2(a) shows that the CS yield increased with temperature when the pH was low and the hydrolysis time was 24 h. When the pH exceeded 6.8, the yield decreased with increasing temperature. The yield was higher when the experimental range of hydrolysis pH and temperature was 6.2–6.8 and 51–58°C, respectively. Compared with the direction A, the curve of the B effect surface was steeper, and the density of the B contour line was significantly higher than that of direction B, indicating that the effect of B on the extraction rate was more significant than that of A.

Figure 2(b) shows that when the enzymolysis temperature was 51°C and the pH was in the acidic range, the CS yield increased with the prolonged enzymolysis time. When the enzymolysis time exceeded 26 h, the yield decreased. The yield was higher when the experimental range of pH and enzymolysis time was 6.2–6.8 and 24–30 h, respectively. The steepness of the response surface curves of A and C was similar, indicating that the effects of A and C on the extraction rate were also similar.

When the pH value was 6.5 and the temperature was relatively low, a longer time was conducive to increasing the CS yield, whereas the yield at higher temperatures decreased with time. The yield was higher when the experimental range of hydrolysis temperature and time was 51–58°C and 24–30 h, respectively (Figure 2(c)). Compared with the direction C, the curve of the B effect surface was steeper, and the density of the B contour line was significantly higher than that of direction B, indicating that the effect of B on the extraction rate was more significant than that of C.

The Design-Expert 12.0 software was used to obtain the optimal enzymatic hydrolysis conditions of CS extracted from Shaanxi cattle nasal cartilage: pH, 6.51; temperature, 64.53°C; and time, 19.86 h. The theoretical yield predicted by the model was 21.62%. The actual average yield was 22.44 ± 0.62%; a difference of 0.82% indicates that the model is suitable for prediction and analysis.

Currently, commercial CS products are mainly derived from shark cartilage and porcine and bovine tissues. The extraction methods for CS include enzymatic, alkaline, neutral salt, and ultrasonic methods. The physicochemical properties of CS prepared using different processes and sources vary [23, 24]. Alkaline hydrolysis involves a *β*-elimination reaction to release CS from proteoglycan. In the alkaline hydrolysis process, the alkali concentration, reaction time, and temperature should be strictly controlled to avoid excessive reactions of the alkali with the carbohydrate chains. In addition, alkaline hydrolysis may cause shedding and glycosyl structural changes due to the nucleophilic attack of the O-sulfuric acid gene on certain GAG glycosyls. The ultrasonic method can greatly shorten the extraction time; however, it simultaneously increases the difficulty of purification, limiting the production of CS. Studies have shown that CS and proteins in the cartilage tissue can be separated at certain ionic strengths in neutral salt solutions of appropriate concentrations. However, the yield obtained by this method is low, and raw materials are wasted, which affects the economic benefits and is not conducive to large-scale production. Nonspecific proteases, such as pronase, pepsin, papain, and trypsin, can hydrolyze glycosaminoglycans into polysaccharides with different peptide segments at the reducing end. Compared with traditional methods, enzymatic extraction requires milder reaction conditions, does not damage the structure of the active components, uses less organic solvents, involves simple procedures, and does not require the use of large equipment [25]. Single enzymes, such as alcalase, trypsin, or papain, are used to hydrolyze the protein for CS isolation. However, a single enzyme hydrolysis step takes at least 24 h, which is not practical for industrial production [26, 27]. Garnjanagoonchorn et al. enzymatically extracted cartilage using papain (4 mg/g of cartilage) and produced a clear solution after 48 h at 65°C. The CS yields from shark fin, crocodile rib, crocodile sternum, and crocodile trachea extracted using this method are 15.05 ± 0.73, 9.05 ± 0.99, 20.09 ± 1.05, and 14.72 ± 1.95, respectively [28]. Ruensodsai et al. extracted CS from *Bohadschia argu0s* and optimized the method using BBD and RSM. The results showed that the CS yield was 4.16 g/100 g dry matter when the papain concentration was 0.48%, the extraction time was 1.01 h, and the extraction temperature was 56.53°C [29]. Rani et al. used papain (1 U/100 mg of tissue) to extract CS with a molecular weight of 100 kDa from chicken breast bones. The reaction condition was 65°C for 24 h, and the yield was 15% [30]. Protein hydrolysis by different proteases has a certain selectivity, and the protein in the CS extract can be more fully hydrolyzed using complex enzymes, which is beneficial for separating and extracting CS. In this study, CS was depolymerized from proteoglycans using the double enzyme method (papain-trypsin), and other impurities were separated by quaternary ammonium salt complex precipitation. This method has the advantages of mild reaction conditions, convenience, rapidity, and no impurities, and it does not easily destroy the CS structure. The extracted CS had high purity and good properties. In this study, the effects of pH, temperature, and time on the CS yield during enzymatic hydrolysis were investigated to develop a cheap and sustainable process using the cattle nasal cartilage which is an agricultural and animal husbandry waste. The RSM was used to optimize the extraction process, which greatly improved the CS yield (21.62%).

### 3.3. Characterization of Physical and Chemical Properties of CS

Different organisms or tissues of the same organism contain different types of CS [28]. Wang et al. purified CS from the skull and spine of large hybrid sturgeon with CPC and produced a yield of 12.95 ± 0.1% and 18.25 ± 0.07%, average molecular weight of 43.9 ± 0.04 and 32.6 ± 0.03 kDa, uronic acid content of 35.72 ± 0.33% and 37.76 ± 0.76%, protein content of 2.87% and 1.96%, and molar ratios of GlcN : GlcUA : GalN : Gal of 0.7 : 4.9 : 4.8 : 1.0 and 0.7 : 4.5 : 4.6 : 1.0, respectively [31]. The molecular weight of CS extracted from chicken cartilage was 100 kDa [30]. Ustyuzhanina et al. separated highly sulfated CS with a molecular weight of 44.1 kDa from the Patagonian sea cucumber *Hemioedema spectabilis* by papain extraction-ion exchange-gel permeation chromatography, and the analysis of monosaccharide and sulfate content of HeSp revealed a GlcUA : GalNAc : Fuc:SO_3_Na molar ratio of 1.15 : 1 : 1.1 : 3.9 [32]. Wang et al. extracted CS1 from chicken leg bone soup by thermal resin static adsorption and CS2 from the end of chicken leg bone by enzymatic method, with average molecular weights of 35.81 kDa and 37.18 kDa, respectively. No evident structural differences were observed in the FTIR spectroscopy and HPLC results [33].

**Figure 3:**
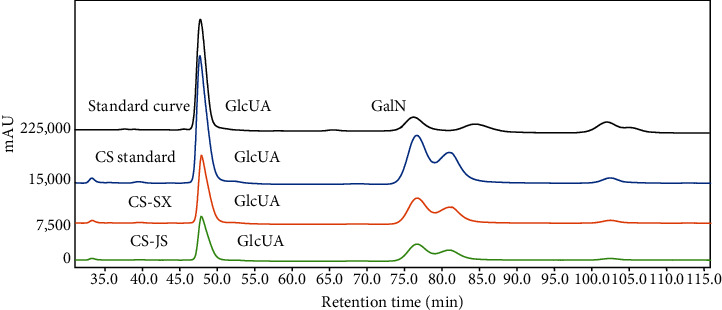
Monosaccharide composition of bovine nose cartilage chondroitin sulfate. CS-SX: CS samples from Shaanxi Yellow cattle nasal cartilage; CS-JS: CS samples from Jiangsu Yellow cattle nasal cartilage.

**Figure 4:**
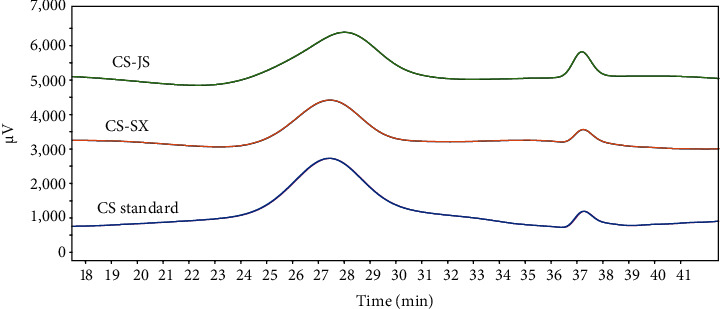
High-performance gel permeation chromatograph of bovine nose cartilage chondroitin sulfate. CS-JS: CS samples from Jiangsu Yellow cattle nasal cartilage; CS-SX: CS samples from Shaanxi Yellow cattle nasal cartilage.

In this study, CS was extracted from bovine nasal cartilage byproducts. The glucuronide, protein, and sulfate group contents are shown in Table 4. No significant differences in glucuronic acid content between CS samples from Shaanxi Yellow cattle nasal cartilage (CS-SX) and Jiangsu Yellow cattle nasal cartilage (CS-JS) were observed. The protein and sulfate contents of CS-JS were slightly higher than those of CS-SX. As shown in Figure 3, CS standard, CS-SX, and CS-JS are mainly composed of GlcUA and GalN, and their molar ratios were 1.39 : 1.0, 1.31 : 1.0, and 1.36 : 1.0, respectively. The unknown peaks might be nondegraded oligosaccharide fragments or products of acid hydrolysis and derivatization. The glycosidic bond of CS was broken after hydrolysis by a strong acid, and the acetyl group was separated from GalNAc to form GalN. The peak emergence time during molecular weight measurement using HPLC is illustrated in Figure 4. The average molecular weights of the CS standard, CS-JS, and CS-SX were 76.90 kDa, 95.52 kDa, and 89.21 kDa, respectively.

**Figure 5:**
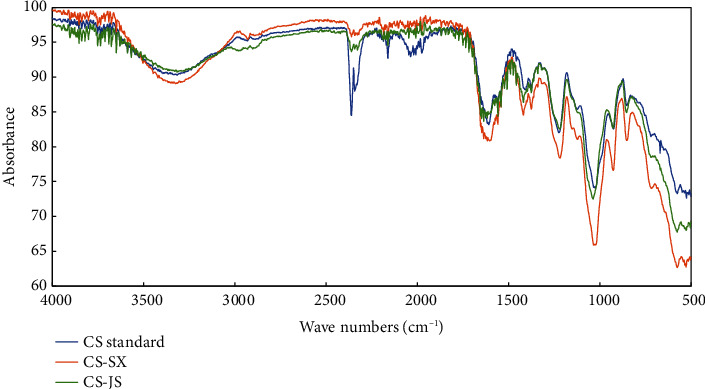
Fourier transform infrared spectra of bovine nose cartilage chondroitin sulfate.

Infrared spectroscopy can maintain the integrity of the sample and provide specific absorption peaks of different functional groups. The spectral wave number and absorption peak positions of the CS samples were consistent with those of the standard (Figure 5), indicating that the extraction method did not destroy the internal structure. Intermolecular and intramolecular hydrogen bonds resulted in broad peaks within the range of 3,500–3,000 cm^−1^, in which O–H and N–H were in telescopic vibration. The weak absorption peak between 2,800 and 3,000 cm^−1^ corresponds to the stretching vibration of the C–H bond; the C=O and N–H groups were between 1,660 and 1,560 cm^−1^, indicating the existence of NH–C–O, a C–N stretching vibration absorption peak at 1,400 cm^−1^, and an acetamide structure. A strong absorption peak at 1,043 cm^−1^ indicated a C–O–S ring, and the prominent absorption peaks of CS-SX and CS-JS at 879 cm^−1^ and 877 cm^−1^, respectively, represented the axial coordination of C–O–S in the C4 sulfate group of galactosamine, validating that CS-A was present in the sample at a high content. The absorption peak at 858 cm^−1^ indicated an S=O bond. No characteristic absorption peak of CS-C was significantly observed at 821 cm^−1^, indicating a very low CS-C content. The presence of some small peaks indicated the presence of impurities in the samples.

Because of its very complex heterogeneous structure, CS from different sources may contain different numbers of disaccharides, as well as sulfate group positions located at different percentages inside the polysaccharide chains. These disaccharide units are generally monosulfated; however, depending on their origin, various disulfated disaccharides (and possibly trisulfated disaccharides) may be present in the carbohydrate backbone. Maccari et al. found that the CS disaccharide composition of monkfish was 28.2% for *Δ*Di6s (*Δ*UA-GalNAc,6s) and 51.0% for *Δ*Di4s (*Δ*UA-GalNAc,4s). The CS disaccharide composition of codfish was 27.3% for *Δ*Di6s and 59.8% for *Δ*Di4s. The CS disaccharide composition of spiny dogfish was 55.7% for *Δ*Di6s and 25.3% for *Δ*Di4s. The CS disaccharide composition of salmon was 37.3% for *Δ*Di6s and 51.2% for *Δ*Di4s. The CS disaccharide composition of tuna (Tuna) was 28.3% for *Δ*Di6s and 63.2% for *Δ*Di4s [34]. Zhou et al. isolated *Raja porosa* CS (RPCS) with a molecular weight of 40,752 Da from *Raja porosa* cartilage using an alkaline protease, and the disaccharides were composed of *Δ*Di6S (CS-C, 65.84%) and *Δ*Di4S (CS-A, 34.16%) [35]. UA-GalNAc4S and UA-GalNAc6S disaccharides in chicken keel bone cartilage CS had percentages of 58% and 42%, respectively [30].

**Figure 6:**
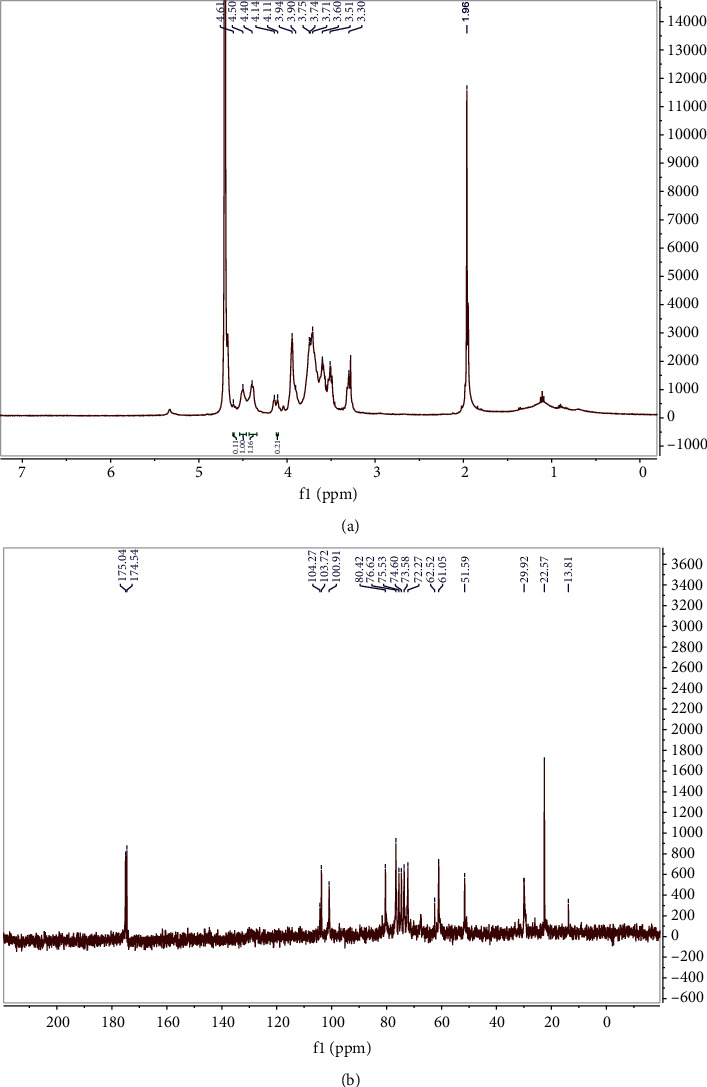
Nuclear magnetic resonance spectra of bovine nose cartilage chondroitin sulfate from Shaanxi cattle: (a) ^1^H-NMR; (b) ^13^C-NMR.

Figure 6 shows the NMR spectra of CS from the bovine nasal cartilage of Shaanxi cattle. These data are consistent with previous literatures [14]. Carbonyl (approximately 175.04 ppm) and acetamido-methyl carbons (22.57 ppm) were observed in the ^13^C NMR spectrum (Figure 6(b)). The signals at 104.27 and 103.72 ppm indicated the presence of GalNAc-6SO_4_ (GlcA-C1) and GalNAc-4SO_4_ (GlcA-C1). The signal at 100.91 ppm indicated C1 of GalNAc. The signal of overlapping peaks between 4.40 and 4.50 ppm in the ^1^H NMR spectrum indicated the H1 of GlcUA and GalNAc. In addition, the signal at 1.96 ppm indicated CH3 protons of GalNAc, and the signals at 4.14, 3.94, and 3.90 ppm were assigned to H4, H2, and H3 of GalNAc. The signal at 3.75 and 3.74 ppm indicated the H5 and H6 of GalNAc, respectively. The signals at 3.30, 3.60, 3.71, and 3.51 ppm were assigned to H2, H3, H4, and H5 of GlcUA, respectively. The characteristic signals at 4.61 and 4.11 ppm were assigned to H4 of GalNAc-4S and H6 of GalNAc-6S (Figure 6(a)).

### 3.4. *In Vitro* Antioxidant Activity of CS

Under normal circumstances, oxidation and antioxidation in the body are in a dynamic balance. When the body is exposed to external stimuli, including environmental pollution, radiation, and excessive stress, the balance is disrupted. The body generates excessive free radicals which attack biological macromolecules such as DNA and proteins, causing serious damage to the body [36, 37].

**Figure 7:**
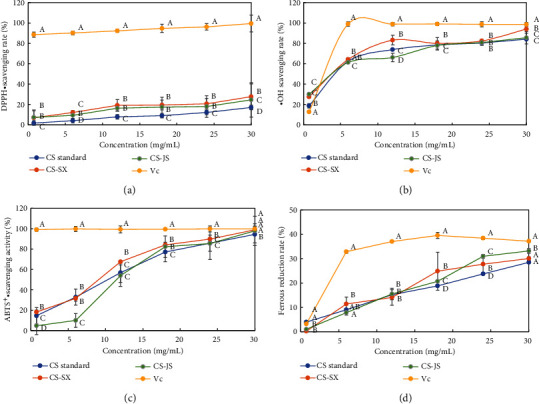
Antioxidant activities of bovine nose cartilage chondroitin sulfate: (a) DPPH• scavenging activity; (b) •OH scavenging activity; (c) ABTS^+^• scavenging activity; (d) Reducing power. Different superscript letters indicate significant differences among samples at the same concentration (*P* < 0.05).

DPPH is a stable free radical that exists as a single-electron molecule. The DPPH alcohol solution is purple. When a sample has free radical scavenging ability, it can be paired with a single electron to cause the solution to fade, and the fading of color is quantitatively related to the number of electrons received. Therefore, the DPPH• scavenging ability of the substrate was determined by measuring the absorbance at 517 nm. The lower the absorbance, the higher the scavenging rate, indicating that the sample had a stronger antioxidant capacity. As shown in Figure 7(a), CS from different sources and concentrations showed good but slightly different DPPH• scavenging abilities. Within a certain range, the scavenging ability increased slowly with increasing concentration, and a significant difference in the DPPH• scavenging ability among the three CS sources was observed. The DPPH• scavenging activities of the 30 mg/mL CS standard, CS-SX, and CS-JS reached 17.14 ± 1.42%, 27.74 ± 3.67%, and 24.57 ± 3.3%, respectively. The scavenging activity of CS-SX was slightly higher than that of CS-JS.

Hydroxyl radicals have extremely strong oxidizing properties and are among the strongest known oxidants [38]. As illustrated in Figure 7(b), both the CS standard and the CS samples showed good •OH scavenging activities. Within the experimental concentration range (0.75–30 mg/mL), the •OH scavenging ability increased with CS concentration. The scavenging ability of CS-SX was slightly stronger than that of CS-JS; however, the difference was not statistically significant. The •OH clearance rates of the 30 mg/mL CS standard, CS-SX, and CS-JS were 84.08 ± 0.25%, 94.13 ± 0.42%, and 85.53 ± 0.30%, respectively. At this concentration, the •OH scavenging rate of the CS standard was not markedly different from that of CS-JS; however, it was significantly different from that of CS-SX (Figure 7(b)).

The ABTS method is often used to test the antioxidant capacities of various substances in vitro. A stable ABTS^+^• solution shows a blue-green color with a maximum absorption at 734 nm, and ABTS^+^• is generated through the reaction of ABTS with K_2_S_2_O_8_. When ABTS^+^• is eliminated, the color of the solution becomes lighter; thus, it can be used to determine the ability of the sample to eliminate ABTS^+^•. As shown in Figure 7(c), within a certain concentration range, the scavenging ability of CS increased with increasing CS concentration. There was no significant difference in the ABTS^+^• scavenging ability between CS-SX and CS-JS. However, there was a significant difference between them and the CS standard. At 30 mg/mL, the clearance rates of CS derived from the standard, Shaanxi, and Jiangsu Yellow cattle against ABTS^+^• were 94.36 ± 1.36%, 98.90 ± 1.75%, and 97.80 ± 1.44%, respectively.

Reducing ability is an important indicator that can be used to determine whether a substrate has antioxidant capacity. As shown in Figure 7(d), Vc exhibited the strongest reducing ability. The CS standard, CS-SX, and CS-JS were all able to reduce ferrous ions, with enhanced reduction ability with increasing CS concentration. The reducing abilities of CS-SX and CS-JS were stronger than that of the standard. The reducing ability of 30 mg/mL CS standard, CS-SX, and CS-JS to ferrous iron was 28.51 ± 0.75%, 30.05 ± 1.07%, and 33.21 ± 0.99%, respectively. The ferrous reduction rate of the CS standard was not markedly different from that of CS-SX. However, it was significantly different from that of CS-JS.

An important factor affecting the biological activity of polysaccharides is their relative molecular weight. Studies have shown that polysaccharides exert optimal activity only when their relative molecular mass (Mr) is within an appropriate range. As Mr increases, the molecular volume and transmembrane resistance of the polysaccharides increase, which impedes absorption and utilization. However, when the relative molecular weight of a polysaccharide is too low, it cannot form an active structure, and its activity decreases [39, 40]. In addition, the antioxidant biological activities of polysaccharides are closely related to the monosaccharide composition, number of functional groups, and connection of the glycosidic bonds [41]. CS is rich in sulfate and carboxyl groups and has a strong negative charge, which may contribute to its strong antioxidant activity [42]. The structural basis of the antioxidant activity of CS is as follows. First, to prevent the production of free radicals, the uronic acid carboxyl and sulfate groups of CS can be chelated with Fe^2+^ and Cu^2+^. Superoxide and H_2_O_2_ combine with transition metal ions to generate highly reactive hydroxyl radicals. Second, the reductive end of CS neutralizes free radicals [43]. The type of disaccharide unit also influences the antioxidant activity of CS, and the difference in antioxidant activity is related to the molecular weight and CS-4 : CS-6 ratio [44]. According to some studies, low-molecular-weight CS (LMWCS) exerts stronger antioxidant activity [14, 45, 46]. The new reducing terminal and unsaturated double bonds produced by enzymolysis can enhance the antioxidant capacity. However, there was no significant difference (less than 5 kDa) in the antioxidant activity of LMWCS, which might be because the unsaturated double bonds and the reducing end of LMWCS were saturated after enzymatic hydrolysis [46]. The excellent antioxidant activity of LMWCS with different terminal structures suggests that its flexible and stretchable conformation enhances its antioxidant activity. Based on the reducibility of the oligosaccharide end groups (aldehyde group > carbon-carbon double bond > ketone group > carboxyl group) obtained by different degradation methods, LMWCS with the same molecular weight prepared by CS cleavage enzyme hydrolysis had stronger antioxidant activity [47]. The Fuc branch of 3,4-S was also more efficient than that of 2,4-S in lowering total cholesterol (TC), LDL, and atherosclerosis indices [48]. In addition, with an increase in the degree of sulfation, the hydrophilicity and structural stability of sugar molecules improved, showing stronger antitumor ability. The extent of branch hypersulfation, especially that of 3,4-S, is critical for anticoagulant activity [49]. Partial hydrolysis of the Fuc branch or removal of the sulfate group greatly reduces its anticoagulant effect [50], possibly because the sulfated and structurally intact fucosylated CS closely matches the thrombin-binding site. In this study, CS extracted from the waste bovine nasal cartilage of Shaanxi cattle showed good antioxidant capacity. Studies have demonstrated that complete chain-length CS cannot penetrate the gastric and intestinal mucosa, whereas LMWCS can penetrate the intestinal mucosa [51]. Thus, a low-consumption, ecological, and controllable hydrolysis method suitable for industrial mass production should be adopted to degrade CS and obtain different LMWCS. The structure-activity mechanism of bovine nasal cartilage LMWCS exerting anti-inflammatory and other functional activities may be explored based on the homeostasis of intestinal flora. Further investigations may be undertaken to achieve high-value utilization of the Yellow cattle and provide a theoretical basis for the comprehensive exploitation of agricultural and animal resources, as well as the development of LMWCS health products.

## 4. Conclusions

This study used RSMs to optimize CS enzymatic extraction while investigating the use of cattle byproducts. The optimal extraction condition was as follows: enzymolysis pH, 6.51; enzymolysis temperature, 64.53°C; and enzymolysis time, 19.86 h. Under these ideal circumstances, an improved CS extraction yield (21.62%) was obtained. The optimized method has obvious advantages, effectively reducing the amount of enzyme and time needed for the extraction, which is conducive to practical application and popularization. FTIR and HPLC results showed that the obtained CS is a medium-molecular-weight polysaccharide with good antioxidant activity. Our results indicate that bovine nasal cartilage is a promising raw material for CS.

## Figures and Tables

**Table 1:** Natural and coded values of independent variables used in the Box–Behnken design (BBD).

Enzymatic hydrolysis process variables	Natural values			Coded values		
	Low	Middle	High	Low	Middle	High
pH (*X*_1_)	5.0	6.5	8.0	-1	0	1
Temperature (*X*_2_, °C)	37	51	65	-1	0	1
Time (*X*_3_, h)	12	24	36	-1	0	1

**Table 2:** Box–Behnken design matrix of variables.

Run^a^	Variable levels^b^			Yield (%)
	*X*_1_	*X*_2_	*X*_3_	Actual
1	5.0	37	24	7.88
2	8.0	37	24	7.70
3	5.0	65	24	13.44
4	8.0	65	24	15.72
5	5.0	51	12	10.51
6	6.5	51	24	21.66
7	5.0	51	36	13.12
8	8.0	51	36	10.13
9	6.5	37	12	5.49
10	6.5	65	12	22.04
11	6.5	37	36	10.19
12	6.5	65	36	14.80
13	6.5	51	24	17.88
14	6.5	51	24	19.19
15	8.0	51	12	7.40
16	6.5	51	24	19.79
17	6.5	51	24	19.41

^a^Experiments were conducted by random order. ^b^*X*_1_: enzymatic hydrolysis pH; *X*_2_: enzymatic hydrolysis temperature (°C); *X*_3_: enzymatic hydrolysis time (h).

**Table 3:** Variance analysis of response surface regression model.

Source^a^	Sum of squares	df	Sum of squares	F value	*P* value
Model	436.63	9	48.51	11.0300	0.0023
*X*_1_	2.00	1	2.00	0.4547	0.5218
*X*_2_	150.86	1	150.86	34.30	0.0006^∗∗^
*X*_3_	0.98	1	0.9800	0.2228	0.6513
*X*_1_ *X*_2_	1.51	1	1.51	0.3440	0.5760
*X*_1_ *X*_3_	0.0036	1	0.0036	0.0008	0.9780
*X*_2_ *X*_3_	35.64	1	35.64	8.10	0.0248^∗^
*X*_1_^2^	133.01	1	133.01	30.24	0.0009^∗∗^
*X*_2_^2^	32.55	1	32.55	7.40	0.0297^∗^
*X*_3_^2^	56.88	1	56.88	12.93	0.0088^∗∗^
Residual	30.79	7	4.40		
Lack of fit	23.35	3	7.78	4.18	0.1002
Pure error	7.44	4	1.86		
Cor total	467.42	16			

^a^ *X*_1_: enzymatic hydrolysis pH; *X*_2_: enzymatic hydrolysis temperature (°C); *X*_3_: enzymatic hydrolysis time (h). ^∗^Significant (*P* < 0.05). ^∗∗^Highly significant (*P* < 0.01).

**Table 4:** Glucuronic acid, protein, and sulfate contents of chondroitin sulfate samples.

	CS standard	CS-SX	CS-JS
Glucuronic acid content (%)	27.19 ± 0.71^a^	31.76 ± 0.72^b^	31.27 ± 0.76^b^
Protein content (%)	0.56 ± 0.08^a^	1.12 ± 0.03^b^	1.74 ± 0.08^c^
Sulfate content (%)	23.83 ± 0.22^a^	23.34 ± 0.08^b^	25.00 ± 0.34^c^

Results were expressed as mean ± SD (*n* ≥ 3). Different superscript letters indicate significant differences among samples (*P* < 0.05).

## Data Availability

The data used to support the findings of this study are included within the article.
